# Assessing the long-term outcomes of telemedicine consultations in managing hypertension: A prospective study

**DOI:** 10.6026/9732063002001904

**Published:** 2024-12-31

**Authors:** Bichu Kuriyan Joy, Shoraf Pascal, Deepika Gnanasekaran, Saranya R., Aswathy Mechur Jayachandran, Arun Sakthivelu

**Affiliations:** 1 Department of Emergency Medicine, Specialist's Hospital, Ernakulam, Kerala, India; 2 Department of Community Medicine, Madha Medical College and Research Institute, Chennai, Tamil Nadu, India; 3 Department of Internal Medicine, Hillingdon Hospital, London, United Kingdom; 4 Department of Community Medicine, Madha Medical College and Research Institute, Chennai, Tamil Nadu, India; 5 Department of General Medicine, Aster Clinic, Dubai, United Arab Emirates; 6 Department of Internal Medicine, PSG Institute of Medical Sciences and Research, Coimbatore, Tamil Nadu, India

**Keywords:** Telemedicine, hypertension, blood pressure control, medication adherence, patient satisfaction, remote healthcare, long-term outcomes

## Abstract

Hypertension is a leading global health concern, contributing significantly to cardiovascular morbidity and mortality. Therefore, it is of interest to assess the long-term effects of telemedicine consultations on blood pressure control, medication adherence and patient satisfaction over one year, comparing it with usual face-to-face care. Results showed that the telemedicine group had significantly better blood pressure control, higher medication adherence (p = 0.002) and greater satisfaction (p < 0.0001) than the control group. Frequent interactions through telemedicine likely facilitated these improvements. Telemedicine demonstrates potential as an effective strategy for managing hypertension in the long term.

## Background:

Hypertension is another name for high blood pressure and it affects more than one billion people. Hypertension accounts for being one of the leading causes of cardiovascular diseases such as stroke, heart failure and kidney disease; therefore, it causes a significant quantity of morbidity and mortality in the world [1]. Proper management of hypertension may lead to prevention of complications eventually. Treatment often includes a combination of lifestyle changes, regular monitoring of blood pressure and adherence to antihypertensive drugs [2]. However, standard management strategies are limited by geographic boundaries, non-availability to medical facilities for health care and time constraints, which often results in the missed regular in-person consultation [3]. This is very many patients with suboptimal blood pressure control and a potential increased risk of cardiovascular complications [4]. Telemedicine is healthcare services provided by the use of telecommunications. This offers a new strategy to all barriers above [5]. It allows for more patient-provider contact, improved health status monitoring and timely modification of treatment plans all without the physical visit to the clinic [6]. Telemedicine can facilitate remote monitoring of blood pressure; improve compliance with medication and personal advice in lifestyle adjustment [7]. From various research studies, it has been established that telemedicine leads to better care of chronic conditions: hypertension, for instance, through increased access and convenience to the services [8]. While promising, the current literature lacks longitudinal impacts of telemedicine in hypertension management [9]. There is a gap in studies that have focused primarily on short-term outcomes. More evidence is needed about sustained impacts of telemedicine over the longer term. The scalability and cost-effectiveness of telemedicine interventions across different healthcare systems would be extremely valuable to investigate [10]. This study will fill this gap by evaluating the long-term consequences of telemedicine consultations in managing hypertension over a period of 12 months. Therefore, it is of interest to document the alteration of blood pressure control and medication adherence, with a comparison being made between telemedicine and traditional in-person care. Findings will provide more information as to whether telemedicine is likely to be feasible as part of routine management of hypertension.

## Methodology:

This prospective study was conducted on January 2023 to December 2023, with a total of 100 adult patients diagnosed with hypertension. The principal aim of this study was to compare telemedicine consultations versus traditional face-to-face consultations for patients with hypertension.

## Inclusion criteria:

[1] Adult patients aged 30 to 70 years and diagnosed with essential hypertension.

[2] Patients who own a home blood pressure monitoring device.

[3] Patients who have internet connectivity to afford telemedicine consultation.

## Exclusion criteria:

[1] Secondary hypertensive patients.

[2] Uncontrolled diabetics or chronic kidney diseases patients.

[3] Patients who are averse or incapacitated to use the telemedicine platforms.

## Study design:

The 100 patients were then divided into two groups.

[1] Group A: Telemedicine Group, where 50 patients received monthly telemedicine consultation and monitored their blood pressure at home.

[2] Group B: Control Group consisted of 50 patients who received classical consultations by visiting the clinic after every three months.

## Telemedicine platform:

The telemedicine group had a safe, HIPAA-compatible electronic platform for video consults, messaging and reporting of blood pressure measurements. Also, the patients were educated on lifestyle changes and medication adherence.

## Data collection:

## Blood pressure monitoring:

Blood pressure was measured using validated home devices. The outcome of interest was systolic and diastolic blood pressure change at the end of 12 months.

## Medication adherence:

Adherence to antihypertensive therapy was assessed with the Morisky Medication Adherence Scale (MMAS-8). This is a widely used, well-validated questionnaire that measures adherence to medication in terms of consistency.

## Patient satisfaction:

Satisfaction about health care services was assessed by using a 5-point Likert scale tool, which inquired into convenience, communication and overall experiences.

## Statistical analysis:

The data were analyzed using SPSS software, version 26. Continuous variables are presented as mean ± SD, while categorical variables are percentages. Changes in blood pressure over time in the groups were compared by paired t-tests while independent t-tests and Chi-square tests were used for outcomes between groups. A p-value of <0.05 was used to determine statistical significance.

## Results:

A total of 100 patients completed the 12-month follow-up. The results are summarized in the following tables. Age, gender distribution, BMI and duration of hypertension were well matched between the groups to allow for balanced baseline study features (Table 1). The telemedicine group had better blood pressure control with a greater change in both systolic and diastolic blood pressure as compared to the control arm (Table 2). The adherence was significantly higher among the telemedicine group as measured by higher MMAS-8 scores (Table 3). The patients who received care in the telemedicine group experienced more convenience and better communication from the initiation to the end of care (Table 4). Patients who received care in the telemedicine group were more consulted by the doctor, with such consultations leading to better follow-up and change in treatment (Table 5). A greater number of patients in the care with telemedicine group adhered more frequently to lifestyle changes and their health improved (Table 6). Fewer emergency visits for hypertension cases were reported in the telemedicine group compared with the control group (Table 7). There were fewer patients in the telemedicine group who needed second-line drugs. It meant that their hypertension was better-controlled (Table 8). The telemedicine group demonstrated greater loss of weight. That reflected better adherence to lifestyle advice (Table 9). The patients in the telemedicine group contacted the health care provider much more often and perhaps that was a reason for better disease management (Table 10).

## Discussion:

The study results show that telemedicine consults are very efficient for the long-term management of hypertension. The patients who have had telemedicine consultations have proved to be responding better in terms of blood pressure control, high adherence to medications and have expressed their satisfaction with care compared to those who had been consulted traditionally in person [11]. These findings are in tandem with prior studies that indicated that clinical outcomes of patients suffering from chronic diseases such as hypertension can be advanced through telemedicine consultation [12]. Telemedicine may provide the potential benefit of a more frequent and flexible interaction between patients and health providers. In the study, telemedicine intervention arm patients had almost threefold consultation than the counterparts in their control arm, thereby allowing for a timely drug and intervention adjustment based on the real-time blood pressure data [13]. This could have resulted in the high improvement of the control of blood pressure in the telemedicine group [14]. Besides greater improved BP, the telemedicine group also showed greater medication adherence, as they had higher MMAS-8 scores. Poor compliance to antihypertensive drugs is one of the major challenges of hypertension management and this often results in uncontrolled blood pressure and increased risk for cardiovascular events [15]. Convenience in accessing of these patients with consultation through telemedicine, along with frequent communication and support from healthcare providers, contributed to greater medication adherence among this group of patients [16]. The patient satisfaction was also highly higher in the telemedicine group, since there is a widespread trend towards the preference of remote healthcare services. Telemedicine is more practical and flexible, because it eliminates the need of patients to travel long distances to be seen at the clinic. Patients receive their care while sitting comfortably at home [17]. It is easier communicating through health providers with digital media platforms, which enhances the patient experience through personalized care responsive to their needs [18]. The study further showed that telemedicine reduced further use of antihypertensive drugs and reduced emergencies to the health care facility, suggesting that telemedicine manages hypertension effectively without progression to more aggressive therapeutic interventions [19]. The findings therefore suggest that telemedicine could be implemented to improve patient outcomes and reduce costs in the treatment of patients with hypertension complicated [20]. Though the concepts and strategies differ according to the degree of development and the government's commitment to offering reasonably priced healthcare services, developing nations are also using telehealth technology [21]. Telehealth is considered to have the potential for the management of hypertension in primary care [22].

## Conclusion:

The study demonstrates that telemedicine consultations significantly enhance long-term hypertension management, resulting in better blood pressure control, improved medication adherence and higher patient satisfaction compared to traditional in-person care. Telemedicine offers a convenient and accessible approach, promoting self-care behaviors and effective disease management. These findings support the integration of telemedicine as a vital component of hypertension management, particularly for patients facing barriers to regular care.

## Figures and Tables

**Table 1:** The baseline characteristics of patients an overview of demographic and clinical parameters in the Telemedicine (A) and Control (B) groups

**Group**	**Age (Mean ± SD)**	**Gender(Male)**	**BMI (Mean ± SD)**	**Hypertension Duration (Years)**
Telemedicine (A)	55.2 ± 8.4	28:22:00	27.6 ± 3.3	6.3 ± 3.4
Control (B)	54.8 ± 8.5	30:20:00	27.4 ± 3.5	6.2 ± 3.6

**Table 2:** Blood pressure control over 12 months

**Group**	**Systolic BP (Mean ± SD)**	**Diastolic BP(Mean ± SD)**	**p-value**
Telemedicine (A)	127.6 ± 10.8	77.9 ± 7.7	
Control (B)	134.8 ± 12.9	81.5 ± 8.1	<0.0001

**Table 3:** Medication adherence among patients

**Group**	**MMAS-8 Score (Mean ± SD)**	**p-value**
Telemedicine (A)	7.1 ± 0.9	
Control (B)	6.5 ± 1.1	0.002

**Table 4:** Patient satisfaction scores

**Group**	**Satisfaction Score (Mean ± SD)**	**p-value**
Telemedicine (A)	4.7 ± 0.3	
Control (B)	4.1 ± 0.5	<0.0001

**Table 5:** The frequency of consultations

**Group**	**Average Consultations per Patient**	**p-value**
Telemedicine (A)	11.6 ± 2.1	
Control (B)	4.3 ± 1.1	<0.0001

**Table 6:** The lifestyle modification adherence

**Group**	**Percentage Adhering to Lifestyle Changes**	**p-value**
Telemedicine (A)	75%	
Control (B)	55%	0.003

**Table 7:** Emergency room visits for hypertension

**Group**	**Number of ER Visits (Mean ± SD)**	**p-value**
Telemedicine (A)	1.1 ± 0.4	
Control (B)	2.2 ± 0.7	0.014

**Table 8:** The use of additional antihypertensive medications

**Group**	**Percentage Requiring Additional Medications**	**p-value**
Telemedicine (A)	18%	
Control (B)	33%	0.039

**Table 9:** The data for weight loss over 12 months

**Group**	**Weight Loss (kg, Mean ± SD)**	**p-value**
Telemedicine (A)	3.3 ± 1.5	
Control (B)	1.9 ± 1.1	0.017

**Table 10:** Frequency of communication with healthcare providers

**Group**	**Frequency of Communication (per month, Mean ± SD)**	**p-value**
Telemedicine (A)	2.7 ± 0.8	
Control (B)	0.8 ± 0.3	<0.0001
